# Optimistic Bias, Risk Factors, and Development of High Blood Pressure and Obesity among African American Adolescents in Mississippi (USA)

**DOI:** 10.3390/ijerph14020209

**Published:** 2017-02-20

**Authors:** Monique S. White, Clifton C. Addison, Brenda W. Campbell Jenkins, Vanessa Bland, Adrianne Clark, Donna Antoine LaVigne

**Affiliations:** 1 Center of Excellence in Minority Health and Health Disparities, School of Public Health, Jackson State University, Jackson, MS 39213, USA; monique.s.white@jsums.edu (M.S.W.); brenda.w.campbell@jsums.edu (B.W.C.J.); donna.antoine-lavigne@jsums.edu (D.A.L.); 2 Jackson Heart Study Community Outreach Center, School of Public Health, Jackson State University, Jackson, MS 39213, USA; misanneone@gmail.com; 3 Jackson Heart Study Graduate Training and Education Center, School of Public Health, Jackson State University, Jackson, MS 39213, USA; nessap1@yahoo.com

**Keywords:** Mississippi, optimistic bias, adolescents, risk factors, high blood pressure, obesity

## Abstract

Childhood obesity has reached epidemic proportions and is linked to hypertension among African American youth. Optimistic bias influences behavior of youth causing them to underestimate their susceptibility to negative health outcomes. This study explored adolescent behaviors and prevalence of high blood pressure and obesity in a school district. We examined the relationship between individual health risk practices and optimistic bias on health outcomes; 433 African American high school students were administered a survey and had their obesity and blood pressure measured by the school nurse. Canonical correlational analyses were used to examine relationships between health risk practices and descriptive statistics for optimistic bias and health outcomes. Engaging in moderate exercise for at least 30 min in the last 7 days and lower blood pressure was the only statistically significant relationship. Two-thirds of the students did not perceive themselves to be at risk of developing cardiovascular disease with males at greater risk than females, despite the presence of clinical risk factors for hypertension and obesity. Reducing health optimistic bias is an effective way of motivating young people to adopt more positive behaviors using educational institutions to implement intervention programs that promote positive health behavior as a way to reduce health disparities.

## 1. Introduction

Cardiovascular disease (CVD) processes begin very early in life and progresses as children become adults [1,2,3]. The prevalence of obesity among children is increasing worldwide and evidence demonstrates that obesity, poor dietary practices, and inadequate physical activity in childhood substantially increase the risk of being an obese adult [4,5,6]. Childhood obesity has also been connected to the incidence of chronic disease in adulthood, such as hypertension and cardiovascular diseases [7,8,9]. 

Some studies have reported significant positive associations between some of the modifiable CVD risk factors and the presence of CVD symptoms [10,11]. By the time many young people are between the ages of 12 years and 19 years, they have already developed risk factors that ultimately place them at an increased risk of developing hypertension [12] and other types of CVD [13]. Children who experience elevated blood pressure are likely to experience elevated blood pressure as adults.

For a very long time, hypertension was believed by many to be an adult onset disease and was not considered to be a major health issue in children. However, the relationship between body weight and blood pressure has become an area of concern, especially since the prevalence of childhood overweight/obesity has increased. With the increase in obesity, children are following the adult obese population, placing themselves at higher risk of becoming obese as adults by continuing to practice negative risk behaviors. There is evidence of an accelerated rate of obesity in children that often tracks into adulthood, where it is a risk factor for many clinical disorders and diseases. Obesity raises the risk for many chronic conditions, including cardiovascular diseases, like diabetes, hypertension, coronary artery disease, and cancer [14,15,16,17]. 

As medical personnel began to define what constitutes normative blood pressure levels in childhood, they began to observe elevated blood pressure in many children and adolescents. Some of these experts have attributed elevated blood pressure in children to the increase of childhood obesity [18]. As childhood obesity increases and awareness of the effects of risk factors increases, the number of children and adolescents diagnosed with hypertension seems to be increasing as well [16]. 

Researchers reported that childhood hypertension was a precursor to hypertension in adulthood, and since hypertension is a possible cause of coronary artery disease (CAD) in adults, hypertension in young people should definitely serve as a warning sign of the future health status of youth [19]. Medical personnel believe that detection and intervention of hypertension at the early stages of development in children are critical to reducing the development of the chronic complications of hypertension [20], since it is generally accepted that severe cases of childhood hypertension can increase the risk of developing other diseases and congestive heart failure [21].

Studies have reported a relationship between blood pressure and body mass index (BMI) [22,23]; black children have higher blood pressure than white children [24,25], and the presence of obesity increases the occurrence of hypertension threefold [26,27].

Optimistic bias (perceived invulnerability or lack of perceived risk) appears to have a greater impact on individuals who struggle to deal with issues that relate to personal control. This situation usually occurs when people overestimate their position or condition in relation to other people [28,29,30] believing that they are at reduced risk of developing diseases than other individuals. This false belief about one’s vulnerability to the multitude of health risk factors could result in at-risk individuals ignoring the preventive actions that could reduce their chances of contracting diseases prematurely [31].

Perceptions of risk for development of negative health outcomes can strongly influence an individuals’ decision to begin a risky practice or behavior and to maintain that pattern for lengthy periods. Studies of adults and adolescents regarding smoking practices have concluded that both groups (adolescents and adults, spurred by optimistic bias, tend to believe that their behaviors will lead to less risk of developing health problems [32]). There is little difference between males and females in their display of optimistic bias and perceived invulnerability [33].

Self-rated health is sometimes used as an indicator of general health [34]. However, research on children and adolescents has shown that there is a mismatch between actual BMI measurements and the children’s and adolescents’ perception of body weight [35]. When adolescents do not realistically perceive the connection between negative behaviors and the initiation and prevalence of poor health, this could contribute to the continuing rise in the prevalence of cardiovascular disease. 

Discrepancies between a medical diagnosis and an individual’s interpretation of his/her own health condition could lead to ineffective treatment of health risk conditions, inadequate prevention strategies, and effective intervention strategies. Lack of agreement between doctors’ health assessments and patients’ interpretation of their health risks could lead to non-compliance that inhibits the identification of health problems and subsequently the application of preventive and remedial plans for reduction or elimination of health risks [36]. Some individuals do not always connect health diagnoses negative risk behaviors they are health optimists who practice optimistic bias. Understanding which group a person belongs to can aid in developing a successful treatment plan for addressing health risks and implementing strategies for prevention and intervention [37].

In 2006, it was estimated that 17.1% of children aged 2 to 18 years, are overweight, and another 16.5% are at risk of overweight [38] who develop a multitude of risk factors that predispose them to developing cardiovascular disease [39]. This study sought to examine the effect of negative behavior practices on the development of high blood pressure and obesity in adolescents in a Mississippi school district, in the context of generalized perceived risk (optimistic bias). Because a person’s opinions, beliefs, and assessments of any situation results in certain types of behaviors [40], the students’ physical activity practices, dietary practices, alcohol consumption, tobacco use, and obesity levels were examined and their association with high blood pressure was assessed. Theoretical health models, like the Health Belief Model [41], Protection Motivation Theory [40], and the Precaution Adoption Process Model [31] are centered on perceived vulnerability; optimistic bias represents a lack of belief of risk for the occurrence of a negative health outcome, they tend to reject protective measures. The cornerstone of cardiovascular health prevention and risk reduction in children and adolescents lies with lifestyle change and the adaptation of healthy behaviors [2].

This study investigated the following hypotheses:

**Hypothesis** **#1:** Health risk behaviors relate to the development of obesity and high blood pressure among adolescents.

**Hypothesis** **#2:** Prevalence of obesity and high blood pressure are related to lack of perceived risk (optimistic bias)?

## 2. Materials and Methods

The sample for this study was 433 students attending one high school in a rural community outside the capital of the State of Mississippi. These students whose ages ranged between 15 and 19 years were enrolled in grades 9 through 12. All students attending the health and physical education classess who agreed to participate in the study, completed the questionnaire. This study used a mixed design using mainly correlational design in addition to qualitative measures to examine the practices of adolescents to determine the level of risk factors that existed and to understand the association between risk behaviors and the development of high blood pressure. Variables measured were the students’ weight, height, health risk behaviors, and risk practices. This study collected primary data, using a questionnaire and the collection of clinical measures, in order to assess the impact of negative student behaviors on their health status. Data and information for evaluating the students’ beliefs, practices, and attitudes were collected through the use of the Student Health History Survey (SHHS). At the completion of the survey, a group of 10 students were randomly selected for a qualitative reflection about what they felt would be effective in motivating students to participate in healthier practices. 

Once approval was granted by the IRB (03-02-10-0345948), and the consent and assent forms were signed, the survey instrument was administered to the subjects. Blood pressure measurements and height and weight measurements were collected by the school nurse. To guarantee measurement accuracy, a Hawksley random-zero sphygmomanometer was used to measure blood pressure. This procedure was completed twice with the students at rest. The students were asked to remove their shoes for the height measurement which was taken using centimeters. This measurement was done using stadiometers constructed by the Medical Instruments Unit of the University of Iowa (Iowa City, IA, USA). Students were asked to wear light clothing to measure their weight. The weight measurement was taken in kilograms by using scales that were calibrated on a weekly basis. Each measurement was made twice and the average of the two measurements was taken. In the event that there was discrepancy in the readings, a third measurement was completed. For this study, students did not complete the measurement portion, until they had a completed survey. The blood pressures were taken by the school nurse. The school had its own procedure in place and accepted responsibility for doing follow-ups when out of range measures should be found.

Data were collected to assess health-risk behaviors such as the level of physical activity performed by students, dietary intake of students, and other health risk practices that have the potential to eventually result in the premature development of chronic diseases. The overarching research questions developed for this study intended to explore to what extent obesity and high blood pressure existed among African American high school children in a rural Mississippi school district in a culture of optimistic bias (perceived invulnerability). The dependent variables for this study were BMI and blood pressure. The independent variables were the health risk behaviors (physical inactivity and dietary intake of fruits and vegetables, tobacco and alcohol use) and optimistic bias of the children examined in this study. Descriptive statistics were computed for all study variables of interest. Values computed were expressed as frequencies and percentages. The relationships between the behavior characteristics of the students, and in BMI and blood pressure measurements were assessed using canonical correlation statistics, through the discriminant analysis procedure to explore the hypotheses and compare the behaviors and practices of these local school students.

### 2.1. Alcohol Consumption

To determine alcohol consumption and tobacco use, the responses of the students on the following questions were examined: (a) Do you drink alcoholic beverages? (b) If yes, identify the drinks you consume; (c) When going out with friends, do you feel it is necessary to drink to have a good time? (d) How much alcohol do you consume on a weekly basis? (e) Do you smoke? (f) If yes, how old were you when you started smoking?

### 2.2. Physical Activity

To determine physical activity and sedentary practices, the responses of the students on the following questions were examined: (a) On how many of past 7 days did you exercise or participate in hard physical activity for at least 30 min (e.g., basketball, soccer, running, swimming laps, fast bicycling, fast dancing, or similar aerobic activities)? (b) On how many of the past 7 days did you participate in moderate physical activity for at least 30 min (e.g., fast walking, slow bicycling, skating, pushing a lawn mower, or mopping floors)? (c) On how many of past 7 days did you do exercises to strengthen or tone your muscles, such as push-ups, sit-ups, or weight lifting? On an average school day, how many hours do you watch TV? (d) In an average week when you are in school, how many days do you go to physical education (PE) classes? (e) During an average physical education (PE) class, how many minutes do you actually spend exercising or playing sports? (f) How many minutes do you actually spend exercising or playing sports? (g) On the average, how many hours of sleep do you get every night?

### 2.3. Dietary Practices

To determine dietary practices, the responses of the students on the following questions were examined: (a) during the past 7 days, how many times did you eat salad? (b) During the past 7 days, how many times did you consume fried foods? (c) How often do you consume soft drinks? (d) How much water do you drink every day? (e) During the past 7 days, how many times did you drink 100% fruit juices such as orange juice, apple juice, or grape juice? Table 1 is a description of the variables for hypothesis testing.

## 3. Results

The mean systolic blood pressure measurement was 130.17 mmHg which is above the recommended level for adults of 120 mmHg (Table 2). The mean diastolic level was 73.53 mmHg which is within the normal range below 80 mmHg. The mean BMI level was 27.37 which is higher than the recommended level (25.0) and puts the average BMI in the overweight category.

A higher percent of the female students (15.2%) were overweight compared to the male students (10.3%). A higher percent of the male students (29.1%) were obese compared to the female students (10.3%). In addition, 12.7% of the students were classified as overweight and 27.0% were classified as obese (Table 3). 

A higher percent of the male students (21.5%) were pre-hypertensive compared to the female students (17.1%). There was no significant difference between gender and hypertension (male students, 3.1%; female students, 2.9%). Over three-quarters of all the students (77.6%) had normal blood pressure, 19.4% were pre-hypertensive, and 3.0% were hypertensive, with more male students classified as pre-hypertensive than females. A higher percent of the students who see themselves as likely to develop cardiovascular disease (19.1%) were underweight compared to students who did not see themselves as likely to develop cardiovascular disease (15.1%). A higher percent of the students who see themselves as likely to develop cardiovascular disease (44.1%) were at normal weight compared to students who did not see themselves as likely to develop cardiovascular disease (39.8%) (Table 4). A higher percent of the students who did not see themselves as likely to develop cardiovascular disease (14.5%) were overweight compared to students who saw themselves as likely to develop cardiovascular disease (11.6%). A higher percent of the students who did not see themselves as likely to develop cardiovascular disease (33.7%) were obese compared to students who saw themselves as likely to develop cardiovascular disease (24.7%). 

There was little difference reported in the mean amount of alcoholic beverages consumed and tobacco used by blood pressure levels (Table 5). The analysis revealed no significant difference in the consumption of alcoholic beverages and the use of tobacco based on blood pressure levels *p* > 0.05.

There was little difference reported in the mean amount of fruits and vegetables eaten (Table 6). The analysis revealed no significant difference in the consumption of fruit and vegetable based on blood pressure levels *p* > 0.05. 

The analysis revealed no significant difference in the amount of physical activity, even though, in general, hypertensive and pre-hypertensive students reported less physical activity (Table 7).

The analysis revealed no significant difference in the amount of amount of tobacco use and alcohol consumption, even though, in general, overweight and obese students reported more tobacco use and alcohol consumption (Table 8).

The analysis revealed no significant difference in the consumption of fruits and vegetables (Table 9).

The analysis revealed no significant difference in the amount of amount of physical activity, even though, in general, overweight and obese students reported less physical activity (Table 10).

As seen in Table 11, 40.5% of the students who were pre-hypertensive believed that it was possible to develop heart disease compared to 59.5% who were pre-hypertensive, but did not believe that it was possible to develop heart disease. In addition, 23.1% of the students who were hypertensive believed that it was possible to develop heart disease compared to 76.5% who were hypertensive, but did not believe that it was possible to develop heart disease.

About 43.6% of the students who were obese believed that it was possible to develop heart disease compared to 56.4% who were obese, but did not believe that it was possible to develop heart disease (Table 12).

About 22.8% of the male students and about 18.4.0% of the female students who think it is possible for them to develop heart disease were classified as pre-hypertensive, while 2.5% of those male students and 1.1% of the female students were hypertensive. On the other hand, 20.8% of the male students and 16.3% of the female students who did not think it is possible for them to develop heart disease were classified as pre-hypertensive, while 3.5% of those male students and 4.1% of the female students were hypertensive Table 13).

The analysis revealed that 34.6% of .the students are predicted to be obese and 24.7% are predicted to be overweight. The data also reveal that 33.7% of the students are predicted to be pre-hypertensive, and 28.2% of the students are predicted to be hypertensive (Table 14).

## 4. Discussion

This study examined two hypotheses related to risk behaviors and heart disease risk factors. The first hypothesis dealt with the relationship between adolescents’ risk behaviors (alcohol use and tobacco consumption, dietary intake of fruits and vegetables, and physical activity) and two risk factors for heart disease, obesity and hypertension. Canonical analyses revealed only one significant finding among those performing moderate physical activity for at least 30 min in the last seven days and lower blood pressure but not for obesity. The second hypothesis dealt with the relationship between optimistic bias and both obesity and hypertension. Although no statistically significant findings were detected through descriptive analyses, two-thirds of the students had optimistic bias in relation to their risk of heart disease; that is, they did not think they could develop heart disease despite a prevalence of obesity, pre-hypertension and hypertension. There was also a nonsignificant pattern of males having more optimistic bias than females.

Behavior risk factors contribute to the development of premature morbidity and mortality. Results from this study demonstrated that this statement could also apply to the health status of high school children in an environment of optimistic bias. Cardiovascular disease processes begin very early in life and progress as children become adults [1,2,3]. However, the majority of students in this study perceived their health status to be good and their chances of developing cardiovascular disease to be minimal. The optimistic appraisal of the students’ health status noted in this study should raise concerns for public health officials and school personnel in view of the degree of risk behaviors that is evident in the school. Previous research has reported about significant associations between some of the preventable behavior risk factors and the presence of CVD symptoms [10,11]. Many researchers have reported on the effect of obesity on the development of for many chronic conditions, such as cardiovascular diseases [14,15,16,17]. A good understanding of the implications of poor choices and negative behavior practices could impact the quality of life for many in addition to reducing the degree of disease development. The results from this study can be used to educate students through improved health and physical education classes whose curriculum can adapt additional activities designed to foster positive behavior practices among the student body. The results may not account for regional differences in obesity rates in other areas in the school district. Since most of the children in the school were required to enroll in health and/or physical education classes, it was expected that a large enough number of students would participate and their responses could give a good indication of the practices of students in that school. However, one has to proceed with caution when considering generalizing the findings to the entire school and all Mississippi school districts. In addition, it is also a challenge to determine whether children can recall accurately what they did at some time in the past. Because behaviors were self-reported, the extent of under-reporting or over-reporting of behaviors could not be determined.

## 5. Conclusions 

The cornerstone of cardiovascular health prevention and risk reduction in children and adolescents is characterized by a combination of lifestyle change and the adaptation of healthy behaviors that can become a major component of the education system curriculum. This is an important first step in the fight to reduce health disparities and health risks that plague many communities around America, and the State of Mississippi [18]. As adult obesity and childhood obesity continue to escalate in Mississippi, education and awareness of the effects of the risk factors are important strategies that would motivate changes in behavior [16]. Some researchers have stressed that inaccurate self-diagnosis can be a predictor of morbidity and mortality [41]. So, it is important for communities to implement strategies that increase the potential for change in health status by implementing targeted person-environment public health interventions to ensure, not only accurate assessment of health status, but also the development of adequate prevention and intervention strategies.

## Figures and Tables

**Table 1:** Description of variables for hypotheses testing.

Variable	Description	Type	Values	Frequency	Percent
High Blood Pressure (mmHg) Diastolic Systolic	Blood pressure measured around the arm above the elbow; it detects the amount of pressure in the blood flowing in the vein (in mm of mercury)	Nominal	1 ≤ 120/80 = Normal	336	77.6
			2 = 120–139/80–89 = Pre-hypertension	84	19.4
			3 = 140+/90+ = High Blood Pressure	13	3.0
BMI (weight in kg/squared height in meters)	Calculated based on calibrated measurements performed by school nurse	Continuous Ordinal	0.0–60++		
			1 ≤ 18.5 Underweight	76	17.6
			2 = 18.5–25.0 Normal	185	42.7
			3 = 26.0–30.0	55	12.7
			Overweight		
			4 ≥ 30.0 Obese	117	27.0
Tobacco Use	Do you smoke	Dichotomous	1 = Yes	63	14.5
			2 = No	370	85.5
Alcohol Use	Do you drink alcoholic beverages?	Dichotomous	1 = Yes	127	29.3
			2 = No	306	70.7
Optimistic Bias	Do you think it is possible that you could develop cardiovascular disease?	Dichotomous	1 = Yes	166	38.3
			2 = No	267	61.7

*n* = 433.

**Table 2:** Mean systolic and diastolic blood pressure and body mass index (*n* = 433).

Blood Pressure	Mean	SD
Systolic Blood Pressure (mmHg)	130.17	19.22
Diastolic Blood Pressure (mmHg)	73.52	9.59
Body Mass Index (kg/m^2^)	27.37	6.53

**Table 3:** Percent distribution of BMI and blood pressure status by gender (*n* = 433).

Outcome	Male	Female	Both
BMI Status			
Underweight	17.5	17.6	17.6
Normal Weight	43.0	42.4	42.7
Overweight	10.3	15.2	12.7
Obese	29.1	24.8	27.0
Blood Pressure Group			
Normal	75.3	80.0	77.6
Pre-Hypertensive	21.5	17.1	19.4
Hypertension	3.1	2.9	3.0

*p* > 0.05.

**Table 4:** Percent distribution of BMI status by optimistic bias (*n* = 433).

BMI Status	Thinks It Is Possible He/She Could Develop Heart Disease		
	Yes	No	All
Underweight	15.1	19.1	17.6
Normal Weight	39.8	44.6	42.7
Overweight	14.5	11.6	12.7
Obese	33.7	24.7	27.0

*p* > 0.05.

**Table 5:** Mean distribution of tobacco use and alcohol consumption by adolescent blood pressure levels (*n* = 433).

Risk Factor	Normal BP Mean (SD) *n* = 77	Pre-Hypertensive Mean (SD) *n* = 185	Hypertensive Mean (SD) *n* = 55	Total Mean (SD) *n* = 433
Drink alcoholic beverages?	1.68 (0.47)	1.70 (0.46)	1.65 (0.48)	1.71 (0.46)
Do you smoke?	1.82 (0.39)	1.85 (0.45)	1.89 (0.31)	1.85 (0.35)

*p* > 0.05.

**Table 6:** Mean distribution of consumption of fruit and vegetables by adolescent blood pressure levels (*n* = 433).

Risk Factor	Normal BP Mean (SD) *n* = 77	Pre-Hypertensive Mean (SD) *n* = 185	Hypertensive Mean (SD) *n* = 55	Total Mean (SD) *n* = 433
Eating fruits and vegetables?	1.29 (0.46)	1.20 (0.40)	1.29 (0.46)	1.23 (0.42)

*p* > 0.05.

**Table 7:** Mean distribution for physical inactivity by adolescent blood pressure levels (*n* = 433).

Risk Factor	Normal BP Mean (SD) *n* = 77	Pre-Hypertensive Mean (SD) *n* = 185	Hypertensive Mean (SD) *n* = 55	Total Mean (SD) *n* = 433
Past 7 days, exercise or participate in hard physical activity at least 30 min?	2.79 (0.98)	2.59 (1.12)	2.47 (1.15)	2.58 (1.12)
Past 7 days, exercise or participate in moderate physical activity for at least 30 min?	2.37 (1.00)	2.36 (1.04)	2.22 (0.98)	2.42 (1.05)
Past 7 days, exercise to strengthen or tone your muscles?	2.58 (0.91)	2.35 (1.08)	2.24 (1.04)	2.40 (1.06)

*p* > 0.05.

**Table 8:** Mean distribution for tobacco use and alcohol consumption by BMI (*n* = 433).

Risk Factor	Underweight Mean (SD) *n* = 77	Normal Mean (SD) *n* = 185	Overweight Mean (SD) *n* = 55	Obese Mean (SD) *n* = 117	Total Mean (SD) *n* = 433
Drink alcoholic beverages?	1.68 (0.47)	1.70 (0.46)	1.65 (0.48)	1.75 (0.43)	1.71 (0.46)
Do you smoke?	1.82 (0.39)	1.85 (0.45)	1.89 (0.31)	1.86 (0.34)	1.85 (0.35)

*p* > 0.05.

**Table 9:** Mean distribution of consumption of fruits and vegetables by BMI (*n* = 433).

Risk Factor	Underweight Mean (SD) *n* = 77	Normal Mean (SD) *n* = 185	Overweight Mean (SD) *n* = 55	Obese Mean (SD) *n* = 117	Total Mean (SD) *n* = 433
Eating fruits and vegetables?	1.29 (0.46)	1.20 (0.40)	1.29 (0.46)	1.20 (0.40)	1.23 (0.42)

*p* > 0.05.

**Table 10:** Mean distribution of physical inactivity by BMI (*n* = 433).

Risk Factor	Underweight Mean (SD) *n* = 77	Normal Mean (SD) *n* = 185	Overweight Mean (SD) *n* = 55	Obese Mean (SD) *n* = 117	Total Mean (SD) *n* = 433
Past 7 days, exercise or participate in hard physical activity at least 30 min?	2.79 (0.98)	2.59 (1.12)	2.47 (1.15)	2.48 (1.18)	2.58 (1.12)
Past 7 days, exercise or participate in moderate physical activity for at least 30 min?	2.37 (1.00)	2.36 (1.04)	2.22 (0.98)	2.66 (1.09)	2.42 (1.05)
Past 7 days, exercise to strengthen or tone your muscles?	2.58 (0.91)	2.35 (1.08)	2.24 (1.04)	2.44 (1.09)	2.40 (1.06)

*p* > 0.05.

**Table 11:** Percent distribution of blood pressure by optimistic bias (*n* = 433).

Blood Pressure	Optimistic Bias Thinks It Is Possible He/She Could Develop Heart Disease	
	Yes (38.3%)	No (61.7%)
Normal	38.4	61.6
Pre-Hypertension	40.5	59.5
Hypertension	23.1	76.7

*p* > 0.05.

**Table 12:** Percent distribution of BMI status by optimistic bias (*n* = 433).

BMI Status	Optimistic Bias Thinks It Is Possible He/She Could Develop Heart Disease	
	Yes (38.3%)	No (61.7%)
Underweight	32.9	67.1
Normal Weight	35.7	64.3
Overweight	43.6	56.4
Obese	43.6	56.4

*p* > 0.05.

**Table 13:** Percent distribution of blood pressure and BMI status by optimistic bias and gender (*n* = 433).

BMI, Blood Pressure	Optimistic Bias					
	Thinks It Is Possible He/She Could Develop Heart Disease			Does Not Think It Is Possible He/She Could Develop Heart Disease		
	(38.3%)			(61.7%)		
	Total	Male	Female	Total	Male	Female
**Blood Pressure**						
Normal	77.7	74.7	80.5	77.5	75.7	79.7
Pre-Hypertension	20.5	22.8	18.4	18.7	20.8	16.3
Hypertension	1.8	2.5	1.1	3.7	3.5	4.1
**BMI Status**						
Underweight	15.1	15.2	14.9	19.1	18.8	19.5
Normal Weight	39.8	39.2	40.2	44.6	45.1	43.1
Overweight	14.5	12.7	16.7	11.6	9.0	14.6
Obese	30.7	32.9	28.7	24.7	27.1	22.0
**Overall Gender**		35.4	41.4		64.6	58.6

*p* > 0.05.

**Table 14:** Predicted BMI and blood pressure groups (*n* = 433).

BMI, Blood Pressure Categories	Number	Percent
BMI Status		
Underweight	100	23.1
Normal Weight	76	17.6
Overweight	107	24.7
Obese	150	34.6
Total	433	100.0
Blood Pressure Group		
Normal	165	38.1
Pre-Hypertensive	146	33.7
Hypertension	122	28.2

*p* > 0.05.
